# Gender Differences in the Application of Spanish Criteria for Initiation of Enzyme Replacement Therapy for Fabry Disease in the Fabry Outcome Survey

**DOI:** 10.3390/ijms17121965

**Published:** 2016-11-24

**Authors:** Miguel-Ángel Barba-Romero, Guillem Pintos-Morell

**Affiliations:** 1 Department of Internal Medicine, Albacete University Hospital, Castilla-La Mancha University, 37 Hermanos Falcó St., 02006 Albacete, Spain; 2 Department of Pediatrics, Germans Trias i Pujol University Hospital and Research Institute (IGTP), Universitat Autònoma de Barcelona, 08916 Badalona, Spain; gpintos.germanstrias@gencat.cat

**Keywords:** agalsidase alfa, enzyme replacement therapy, Fabry disease, Fabry Outcome Survey (FOS), gender differences, Spain, women

## Abstract

Both male/female patients with Fabry disease (FD) may receive enzyme replacement therapy (ERT). Previously published analyses of the Fabry Outcome Survey (FOS; Shire-sponsored) database suggested gender differences in timing of ERT initiation. We assessed alignment of criteria for ERT initiation in the Spanish adult population included in FOS with recommendations of a Spanish national consensus. This retrospective analysis examined baseline clinical data of 88 adults (49 females) enrolled in the FOS database up to August 2014. Thirty-five (39.8%) patients were not receiving ERT: five (12.8%) males and 30 (61.2%) females. Baseline disease severity on the FOS-derived Mainz Severity Score Index was lower in untreated males (median (interquartile range), 0.0 (0.0–1.0)) than treated males (TM; 15.0 (7.5–26.5)), and was similar in untreated and treated females. The percentage of untreated females with at least one criterion for treatment initiation was 76.7% versus 100.0% of treated females (*p* = 0.0340) and 97.1% (*p* = 0.0210) of TM. In discordance with Spanish consensus recommendations, a substantial number of females with evidence of FD who might benefit from ERT have not yet initiated treatment. These results suggest unequal gender perceptions with respect to ERT initiation in Spain.

## 1. Introduction

Fabry disease (FD) is a rare inherited X-linked metabolic disease secondary to reduction/absence of lysosomal α-galactosidase A activity. As a result, a progressive accumulation of globotriaosylceramide (Gb_3_) and related glycosphingolipids within lysosomes is believed to produce cellular changes that progressively affect multiple organ systems, determining a natural disease evolution ranging from an asymptomatic status in the first years of life to different clinical presentations with increasing age. FD in adults has a wide variety of phenotypes, from the “classical” severe form in males to a seemingly asymptomatic course in some females. Owing to the X-linked nature of the disease and the potential for skewed X-chromosome inactivation, females can have normal α-galactosidase A activity in plasma/leukocytes with variable signs and symptoms of FD [1,2]. Most heterozygous females develop symptoms with vital organ involvement, usually later than males [1]. FD manifestations may include neuropathic pain, gastrointestinal disturbances, angiokeratomas, hypohidrosis, kidney dysfunction, cardiac valve disease, cardiomyopathy and stroke, resulting in a reduction of health-related quality of life (HRQoL) and an increased risk of premature mortality [3].

Treatment of FD in male/female (pediatric and adult) patients with enzyme replacement therapy (ERT) has been shown to stabilize progressive multiorgan decline and improve clinical outcomes [1,4,5,6]. ERT reduces plasma and urine Gb_3_ and lyso-Gb_3_ levels, ameliorates early clinical symptoms such as pain and gastrointestinal symptoms and improves heart rate variability and HRQoL [7,8,9,10]. At the organ level, ERT reduces left ventricular mass (LVM) and ventricular wall thickness, and slows the progression, or stabilizes, mild to moderate nephropathy as assessed by estimated glomerular filtration rate (eGFR) [11,12,13,14,15]. Indeed, the pattern of mortality has changed since the introduction of ERT, from a higher percentage of deaths by renal failure in males and cerebrovascular disease in females, to cardiac disease in both genders [16]. Although few studies have been published specifically describing ERT effects in female patients with FD [4,17,18,19], a direct comparison of agalsidase alfa ERT effectiveness between male and female patients using data from the Fabry Outcome Survey (FOS) showed “that women are as likely to respond to ERT as men” [20].

Several expert panel–derived guidelines for initiation of ERT have been proposed on the basis of published evidence for efficacy and local health care system variations [21,22,23]. In Spain, a 2005 national consensus document set criteria for initiation of ERT in patients with FD independent of patient gender [24]; an update of this became available in 2011 [25].

FOS, sponsored by Shire, is a global international multicenter registry of patients with a confirmed diagnosis of FD who are receiving, or are candidates for, ERT with agalsidase alfa. Previously published analyses of Spanish patients included in the FOS database suggested gender differences at the time of ERT initiation [26,27]. The aim of our research was to assess the extent to which criteria for ERT initiation in the Spanish adult population included in FOS align with recommendations of a national consensus document.

## 2. Results

In August 2014, 88 Spanish adult patients from 28 hospital centers were included in FOS, of whom 49 (55.7%) were female. Overall median (interquartile range (IQR)) for age at symptom onset was 14 (10–25) years in males and 27 (16–39) years in females.

A total of 53 (60.2%) patients were receiving ERT, including 87.2% of males and 38.8% of females. The groups studied comprised 34 treated males (TM; 38.6% of the total sample; 87.2% of all males), five untreated males (UM; 5.7% of the total sample; 12.8% of all males), 19 treated females (TF; 21.6% of the total sample; 38.8% of all females) and 30 untreated females (UF; 34.1% of the total sample; 61.2% of all females). Baseline clinical characteristics are shown in Table 1.

Males and females receiving ERT at baseline began treatment at a median (IQR) age of 41.4 (33.2–50.0) years; age at treatment initiation was independent of gender, even when age at symptom onset was different. Median (IQR) age of symptom onset was lower in TM (14.0 (10.0‒25.0) years) than in TF (30.5 (16.0‒41.0) years; *p* = 0.027). The median (IQR) age at treatment initiation in TF, 47.7 (35.8–52.8) years, was not significantly different than the median (IQR) age at data extraction in UF, 45.7 (35.4–57.5) years (*p* = 0.910). FOS-Mainz Severity Score Index (MSSI) scores of treated male patients were higher than those of untreated male patients, indicating greater disease severity in treated versus untreated male patients. The median baseline FOS-MSSI score was not significantly different between treated and untreated female patients. HRQoL, as measured by EuroQol 5-Dimensions (EQ-5D), was assessed for only 3 of 30 (10%) UF, 6 of 19 (32%) TF and 11 of 34 (32%) TM.

The percentage of patients with proteinuria (recorded as “signs and symptoms” in the FOS database; Table 1) was much lower in UF (16.7%) compared with TM (61.8%; *p* < 0.0001), and was approximately half that seen in TF (36.8%; *p* = 0.1730). Analytical values for proteinuria (Table 1) were present in 25.0% of UF, versus 70.6% (*p* = 0.0250) of TM and 54.5% (*p* = 0.2140) of TF. The percentage of patients with eGFR <90 mL/min/1.73 m^2^ was 30.0% in UF, 36.8% in TF, 0% in UM and 52.9% in TM (Table 1). Baseline microalbuminuria with a renal biopsy suggestive of FD was seen in 1 UF, 1 TF, 2 TM and in no UM. Left ventricular hypertrophy (LVH) recorded as “signs and symptoms” in the FOS database was present in 36.8% of TF and 23.3% of UF (*p* = 0.3460).

**Figure 1:**
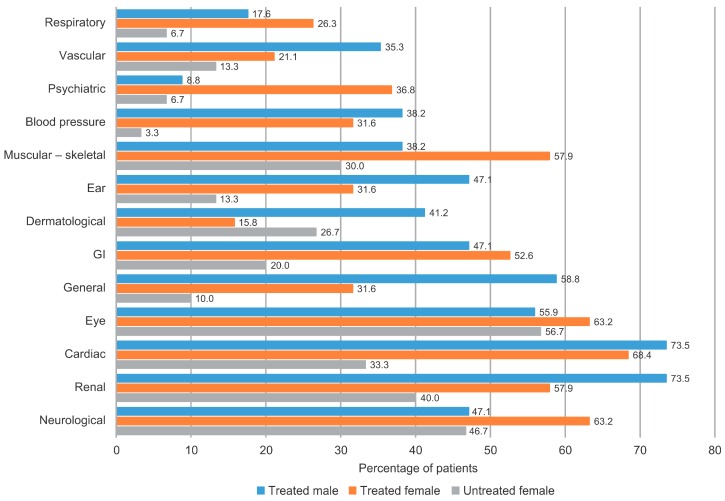
Comparison of patients by organs affected in treated males and females and untreated females. FOS, Fabry Outcome Survey; GI, gastrointestinal.

UF did not substantially differ from treated patients in disease characteristics such as pain or other markers of renal and cardiac involvement. Neuropathic pain was present in 30.0% of UF, with a distribution of median (IQR) Brief Pain Inventory (BPI) scores for worst (8.0 (8.0–8.0)), least (2.5 (0.0–5.0)) or average (3.5 (0.0–7.0)) pain during the previous 24 h, or for pain intensity at the visit (7.5 (7.0–8.0)) that did not notably differ from those in the other groups of patients. Median (IQR) EQ-5D index score in UF (0.8 (0.7–1.0)) was similar to that in TF (0.7 (0.3–0.7)). A graphical comparison of the proportion of patients by organs affected in TM, TF and UF is displayed in Figure 1.

As a result, the percentage of UF fulfilling at least one criterion of the Spanish guidelines for treatment initiation was 76.7%, which differed from the percentage of both TF (100%; *p* = 0.0340) and TM (97.1%; *p* = 0.0210; Table 2). The presence of other criteria was as follows: 30.0% of UF met pain criteria (versus 47.4% of TF (*p* = 0.2420) and 41.2% of TM (*p* = 0.4370)) and 23.3% of UF met cardiac criteria (versus 52.6% of TF (*p* = 0.0630) and 55.9% of TM (*p* = 0.0110)). Further, 43.3% of UF met renal criteria (versus 57.9% of TF (*p* = 0.3870) and 82.4% of TM (*p* = 0.0020)). Distribution of patients according to the criteria is shown in Table 2. 

## 3. Discussion

The study of rare diseases is intrinsically impaired by their low prevalence, making a complete picture of clinical characteristics and management difficult to ascertain. Therefore, global multicenter registries are an essential tool for the study of rare diseases; the FOS registry is one of the largest disease-specific registries of patients with FD. Although inter-center variations in data collection procedures are common, they are addressed by a common protocol that defines the standard data to be collected and unifies them into a single database for analysis. The noninterventional nature of this research method permits the observation of clinical practice variability in management of FD that may lead to detection of unmet needs and disparities or divergences from current recommendations. This paper describes a set of Spanish adult patients at inclusion in FOS according to treatment status and gender. One of the objectives was to present clinical characteristics of this population and to compare the treatment status with the recommendations from a national consensus panel to explore deficiencies in ERT access.

The relatively short period since ERT introduction in 2001, the low prevalence of FD and its slow progression hampers understanding of long-term and patient-related outcomes and mortality. Consequently, international guidelines for ERT initiation are mostly based on expert panel recommendations. As a result, guidelines vary from one country to another, particularly in management of heterozygous females and children [21]. Spanish guidelines existing at the time when the patients entered FOS did include the definitions and criteria from international guidelines, but did not differentiate patients by age or gender regarding when it should be advisable to start ERT [24]. However, the presence of one major criterion in females is consistent with the international consensus document regarding indications for ERT in females when progression of organ involvement is detected [23]. Spanish guidelines were updated in 2011 [25], maintaining the main criteria to start ERT, but refining some definitions and indications for treatment initiation in light of international consensus. In our opinion, the fact that the main criteria to start ERT were the same in the 2011 update as those proposed in 2005, but were more thoroughly defined and less vague in the update, may have increased the number of patients deemed as qualifying for ERT, but without any relevant effect on the number of patients not receiving ERT, especially females; this is one of the most striking findings of our work.

The gender differences observed in access to ERT according to existing guidelines confirm that a proportion of female patients with FD fulfill criteria for ERT initiation, but are not receiving treatment. This finding has previously been suggested in published analyses of the Spanish patients in FOS [26,27], all patients in FOS [20] and other studies from different registries [28,29,30,31,32]. Disease rarity, misconceptions about their carrier status and gender have been proposed as the main drivers for the differential access to ERT for females with FD [33]. Evolving knowledge of the natural history of FD and its management with ERT has changed the consideration of heterozygous females from obligate carriers, mostly asymptomatic or with mild disease, to patients with important clinical features and time-dependent disease progression without ERT [30,34,35]. Evidence from the literature suggests that females are referred less often for diagnostic interventions and treated less aggressively than males [33]. Furthermore, disparities in treatment between genders have been consistently identified for heart [36] and kidney diseases [31,37], among others [32].

The fact that there were no differences in the number of affected organs between UF and TF or between UF and TM suggests the multisystem nature of the disease in both genders in our patients. There was no significant difference between UF and TF in FOS-MSSI scores, and 76.7% of UF fulfilled at least one criterion for ERT initiation. Considered independently, 60.0% of UF fulfilled one renal criterion, most frequently renal impairment (eGFR < 90 mL/min/1.73 m^2^ in 30.0%). This is similar to values reported for UF in other registries (e.g., 55% in Ortiz et al.) [28], and similar or slightly lower than those reported in the literature for combined TF and UF (e.g., 58% in Wang et al. [30], 62.5% in Wilcox et al. [38]). Proteinuria (recorded as a sign/symptom) was observed in 17% of UF, similar to the 16% reported for the whole group of Spanish females included in FOS in 2009 [26]. A recent review noted that progressive nephropathy is prominent in FD and although males are more profoundly affected than females, the authors concluded that both males and females should initiate ERT if they have evidence of renal involvement [39]. More than half of UF met the criteria to start ERT based on cardiac involvement. These included LVM index as the major driver, atrioventricular block and LVH. The observed LVH frequency does differ from some reports for all females in previous publications [30,34]; however, prevalence of LVH is age dependent and is higher in older populations. Recently, Hopkin et al. presented data from the Fabry Registry showing that delayed ERT, as well as having experienced a previous clinical event (cardiac, renal or cerebrovascular) before ERT start, are risk factors for an unfavorable evolution and the appearance of new clinical events under ERT, in male and female patients alike [40]. In accordance with this, the absence of timely ERT initiation in female as well as in male patients showing cardiac or renal involvement of FD may jeopardize their clinical evolution. This clearly applies to patients with classic FD; however, atypical milder, later-onset phenotypes have been associated with variant mutations, including cardiac and cerebrovascular variants [41,42,43,44].

Recently, Lenders et al. published the findings of a multicenter German study with 224 genetically confirmed adult female patients with FD [45], investigating their current ERT status at the time of their last visit to analyze whether patients were treated in accordance with current European FD guidelines (class I and IIA/B recommendations) [23]. It is noteworthy that these recommendations are quite similar to the 2011 update of the Spanish recommendations [25]. Lenders et al. found in their cohort that one-third of German females without ERT fulfilled indications for starting it [45]. Unlike the population in the German cohort where TF were older than UF, in our patients we did not see any differences in age between UF at data collection and TF at ERT start. In addition, in our patients as well as in the German cohort, both TF and UF showed a significant number of organs affected by the disease as an expression of multisystemic involvement. Moreover, the main organ manifestations seen in German TF were cardiac and, to a lesser extent, renal [45]; similarly, in our Spanish TF patients, cardiac involvement was somewhat more frequently seen than renal involvement. It thus appears that there are some differences in the management of female compared with male patients with FD in clinical practice in Germany as well as in Spain.

Two limitations of our research should be mentioned. First, the numbers of patients overall, and especially in the various subgroups, are quite small, with missing data for some parameters. The data were collected from an observational registry that was not specifically designed to assess gender differences in ERT initiation. Together with the large number of evaluated outcomes, these factors might have reduced the power and robustness of the statistical tests. Selection bias is a recognized limitation of registry studies and not all patients in our sample had complete data, a reflection of real-world clinical practice.

The second limitation of our study could be the use of ERT initiation criteria according to an updated version of the 2005 recommendations. This update might have increased the sensitivity of cardiac and, to a lesser extent, renal criteria for detecting treatment candidates and, consequently, may have modified the classification for a certain number of patients. It should be noted that these data were collected more than three years after the last recommendations update; therefore, physicians should have had enough time to adopt the latest criteria. In our opinion, failure to do so reflects nonadherence to recommendations regardless of the proposed criteria, rather than the effect of other factors. According to our current knowledge, some female patients with mild renal involvement (e.g., with microalbuminuria and a slight decrease in eGFR to 80–90 mL/min/1.73 m^2^) could have slow clinical progression and without objective signs of other organ damage (cardiac, central nervous system, pain or gastrointestinal symptoms), a personalized approach is warranted and ERT could be delayed with careful and continuous follow-up. Additionally, some of the assessments were not available for all patients (e.g., laboratory results for proteinuria, some echocardiographic assessments and EQ-5D or BPI score).

Nonetheless, from our point of view, the current study is important because it addresses differences based on gender regarding ERT initiation in Spain, somewhat similar to that observed in another European country. As with many diseases, clinicians in the real-world setting derive their best practices from both clinical practice guidelines and their own clinical experience and impressions. There is an ongoing need in the medical community for greater and more widespread knowledge regarding FD and other rare diseases to illuminate our current understanding that heterozygous females with FD may have substantial disease effects. In turn, this can only enhance our efforts to offer the best standard of care for both men and women with FD. Disease registry analyses, such as the current one, can offer valuable insights into real-world disease management.

## 4. Materials and Methods

### 4.1. Design

This was a retrospective analysis of baseline clinical data of adult patients with FD who were managed in Spanish centers and enrolled in the FOS database up to August 2014.

### 4.2. Study Population

Characteristics of the FOS registry that started data collection in 2001 have been described elsewhere [46,47]. Briefly, the FOS registry collects standardized information from patients who are managed at participating centers and provide signed informed consent. FOS has been approved by the ethics committee/institutional review board of all participating centers and all procedures were in accordance with the Declaration of Helsinki of 1975, revised in 2013. Information obtained during routine clinical follow-up includes baseline and clinical laboratory data plus additional information on patient-reported outcomes collected through questionnaires (e.g., pain and HRQoL) [46]. Additionally, disease severity is assessed through the FOS-MSSI, an adaptation of the MSSI to a binary format data input [48,49].

### 4.3. Study Measures

The 2005 Spanish national consensus document stated that ERT should be initiated for FD immediately upon presentation of any one of the following signs or symptoms (major criteria) [24]: severe neuropathic pain, nephropathy (proteinuria >300 mg/24 h in adults or >5 mg/kg/24 h in children; eGFR < 80 mL/min/1.73 m^2^; renal biopsy), cardiac disease (LVH, ischemic heart disease or arrhythmias) or cerebrovascular disease (clinical or neuroradiological signs). Additionally, ERT initiation may be considered when at least two of the following FD symptoms are present (minor criteria): hypoacusia or vertigo interfering with HRQoL, gastrointestinal manifestations, asthenia, episodic fever, osteoarticular disease, growth delay, microalbuminuria or mild acroparesthesia. In the 2011 update, the former criteria were refined regarding main organ involvement, and it was emphasized that such criteria should be the same for every patient regardless of gender [25]. Specifically, the eGFR criterion was raised to 90 mL/min/1.73 m^2^, more specific electrocardiographic and echocardiographic criteria were considered for diagnosis of cardiac involvement and microalbuminuria was “upgraded” from a minor to a major criterion, but with a renal biopsy with FD findings [25].

eGFR was calculated with serum creatinine values adjusted (if necessary) by the analytical method used at each center to achieve uniformity [50]. LVH was considered when LVM index was ≥51 g/m^2.7^ in males or ≥48 g/m^2.7^ in females. LVM index was determined by standard M-echocardiography at each participating center and adjusted for height using the Devereux formula [51]. HRQoL was assessed through the EuroQol Group’s measure of health status (EQ-5D) [52], using a descriptive system of five categorical dimensions, a visual analog scale ranging from 0 (death) to 100 (full health) and a derived tariff based on population weights from 0–1 with the same extreme anchors [53]. The MSSI consists of four sections covering various signs and symptoms of the disease (general, neurological, cardiovascular and renal), weighted in accordance with their contribution to morbidity. Hence, a global score was obtained to enable patient classification according to disease severity.

### 4.4. Statistical Analyses

Descriptive and analytical analyses were performed for the overall sample and for subgroups created according to gender and ERT status: TM, TF, UM and UF. Categorical variables were described by their frequency and percentage. Continuous variables were described by median and IQR (IQR = Q1 − Q3). Comparisons between two independent samples were made with Wilcoxon rank-sum test for continuous variables and Fisher’s exact test for categorical variables.

## 5. Conclusions

These results suggest gender differences in initiation of ERT in Spain. With reference to recommendations of a Spanish consensus on FD, a substantial number of females with evidence of FD may benefit from ERT but have not yet initiated treatment. The ERT initiation delay in female patients who fulfill the criteria for ERT initiation results in these patients missing the full benefits of treatment and might put them at risk of FD complications with associated morbidity and mortality.

## Figures and Tables

**Table 1:** Baseline clinical characteristics of Spanish patients with Fabry disease included in the FOS registry as of August 2014 by gender and treatment status.

Characteristic	All	All Treated	TM	TF	UF	UM
*n* (%) ^1^	88 (100.0)	53 (60.2)	34 (38.6)	19 (21.6)	30 (34.1)	5 (5.7)
Median (IQR) age at symptom onset (years)	20.0 (11.0–33.5) *n* = 48	20.0 (10.0–35.0) *n* = 37	14.0 (10.0–25.0) ^2^ *n* = 23	30.5 (16.0–41.0) *n* = 14	25.0 (16.0–32.0) *n* = 11	0 *n* = 0
Median (IQR) age at data extraction (years)	46.6 (36.0–54.8) *n* = 88	47.1 (41.1–54.4) *n* = 53	46.1 (38.2–52.1) *n* = 34	53.1 (42.8–64.9) *n* = 19	45.7 (35.4–57.5) *n* = 30	33.1 (25.5–34.5) *n* = 5
Median (IQR) age at ERT initiation (years)	—	41.4 (33.2–50.0) *n* = 53	38.3 (30.2–46.6) *n* = 34	47.7 (35.8–52.8) *n* = 19	—	—
Median (IQR) total FOS-MSSI score	9.5 (5.3–16.5) *n* = 88	14.0 (7.0–20.0) *n* = 53	15.0 (7.5–26.5) ^3^ *n* = 34	11.0 (6.0–17.0) *n* = 19	8.0 (4.5–10.0) *n* = 30	0.0 (0.0–1.0) *n* = 5
Median (IQR) EQ-5D index score	0.7 (0.5–0.8) *n* = 20	0.7 (0.3–0.8) *n* = 17	0.8 (0.3–0.8) *n* = 11	0.7 (0.3–0.7) *n* = 6	0.8 (0.7–1.0) *n* = 3	– *n* = 0
Median (IQR) number of organs affected	4.0 (3.0–6.5) *n* = 84	5.0 (3.0–8.0) *n* = 53	5.5 (3.0–8.0) ^3^ *n* = 34	5.0 (3.0–8.0) ^3^ *n* = 19	4.0 (2.0–4.0) *n* = 28	2.0 (1.0–3.0) *n* = 3
Neuropathic pain, *n* (%) ^1^	32 (36.4)	23 (43.4)	14 (41.2)	9 (47.4)	9 (30.0)	0
eGFR < 90 mL/min/1.73 m^2^, *n* (%) ^1^	34 (38.6)	25 (47.2)	18 (52.9)	7 (36.8)	9 (30.0)	0
Proteinuria: signs or symptoms, *n* (%) ^1^	34 (38.6)	28 (52.8)	21 (61.8) ^3^	7 (36.8)	5 (16.7)	1 (20.0)
Proteinuria > 300 mg/24 h, *n* (%) ^1^	21 (51.2)	18 (64.3)	12 (70.6) ^3^	6 (54.5)	3 (25.0)	0
Dialysis, *n* (%) ^1^	7 (8.0)	7 (13.2)	7 (20.6) ^2,3^	0	0	0
Renal transplant, *n* (%) ^1^	7 (8.0)	7 (13.2)	7 (20.6) ^2,3^	0	0	0
LVH: signs or symptoms, *n* (%) ^1^	33 (37.5)	25 (47.2)	18 (52.9) ^3^	7 (36.8)	7 (23.3)	1 (20.0)
LVH based on LVM index ≥48 g/m^2.7^ (females) or ≥51 g/m^2.7^ (males), *n* (%) ^1^	15 (50.0)	13 (61.9)	10 (71.4) ^3^	3 (42.9)	2 (22.2)	0
Atrioventricular block, *n* (%) ^1^	1 (1.1)	1 (1.9)	0	1 (5.3)	0	0
Bundle branch block, *n* (%) ^1^	3 (3.4)	2 (3.8)	2 (5.9)	0	1 (3.3)	0
Arrhythmia, *n* (%) ^1^	4 (4.5)	4 (7.5)	4 (11.8)	0	0	0
Transient ischemic attack, *n* (%) ^1^	2 (2.3)	2 (3.8)	1 (2.9)	1 (5.3)	0	0
Stroke, *n* (%) ^1^	2 (2.3)	2 (3.8)	1 (2.9)	1 (5.3)	0	0

eGFR, estimated glomerular filtration rate; EQ-5D, EuroQol 5-Dimensions; ERT, enzyme replacement therapy; FOS, Fabry Outcome Survey; IQR, interquartile range; LVH, left ventricular hypertrophy; LVM, left ventricular mass; MSSI, Mainz Severity Score Index; TF, treated female; TM, treated male; UF, untreated female; UM, untreated male; ^1^ Percentage of total patients assessed; ^2^ Statistically significant difference versus TF (*p* < 0.05); ^3^ Statistically significant difference versus UF (*p* < 0.05).

**Table 2:** Description of the distribution of Spanish patients included in the FOS registry in August 2014 according to the criteria for treatment initiation stated in the Spanish national consensus of 2005 [24], updated in 2011 [25], by gender and treatment status.

Treatment Criteria	All	All Treated	TM	TF	UF
*n* (%) ^1^	88 (100.0)	53 (60.2)	34 (38.6)	19 (21.6)	30 (34.1)
Fulfilling ≥1 criteria from all parameters, *n* (%)	76 (86.4)	52 (98.1)	33 (97.1) ^2^	19 (100.0) ^2^	23 (76.7)
Pain criteria	32 (36.4)	23 (43.4)	14 (41.2)	9 (47.4)	9 (30.0)
Renal criteria	53 (60.2)	39 (73.6)	28 (82.4) ^2^	11 (57.9)	13 (43.3)
Cardiac criteria	37 (42.0)	29 (54.7)	19 (55.9) ^2^	10 (52.6)	7 (23.3)
Neurological criteria	2 (2.3)	2 (3.8)	1 (2.9)	1 (5.3)	0

FOS, Fabry Outcome Survey; TF, treated female; TM, treated male; UF, untreated female; ^1^ Percentage of total patients; ^2^ Statistically significant difference versus UF (*p* < 0.05).
